# Supplementary material for: Bayesian refinement of protein structures and ensembles against SAXS data using molecular dynamics
Source: PLoS Comput Biol. 2017 Oct 18;13(10):e1005800. doi: 10.1371/journal.pcbi.1005800 (PMC5662244; doi:10.1371/journal.pcbi.1005800)
Supplement: S1 Archive — (BZ2) [file pcbi.1005800.s011.tar.bz2 › gromacs_4.62_bayesian_saxs_refi/share/html/online/g_rmsf.html]

g\_rmsf


| g\_rmsf Main Table of Contents |

**VERSION 4.6  
Sat 19 Jan 2013**

---

### Description

g\_rmsf computes the root mean square fluctuation (RMSF, i.e. standard
deviation) of atomic positions in the trajectory (supplied with -f)
after (optionally) fitting to a reference frame (supplied with -s).

With option -oq the RMSF values are converted to B-factor
values, which are written to a .pdb file with the coordinates, of the
structure file, or of a .pdb file when -q is specified.
Option -ox writes the B-factors to a file with the average
coordinates.

With the option -od the root mean square deviation with
respect to the reference structure is calculated.

With the option -aniso, g\_rmsf will compute anisotropic
temperature factors and then it will also output average coordinates
and a .pdb file with ANISOU records (corresonding to the -oq
or -ox option). Please note that the U values
are orientation-dependent, so before comparison with experimental data
you should verify that you fit to the experimental coordinates.

When a .pdb input file is passed to the program and the -aniso
flag is set
a correlation plot of the Uij will be created, if any anisotropic
temperature factors are present in the .pdb file.

With option -dir the average MSF (3x3) matrix is diagonalized.
This shows the directions in which the atoms fluctuate the most and
the least.

### Files

| option | filename | type | description |
| --- | --- | --- | --- |
| **-f** | traj.xtc | Input | Trajectory: xtc trr trj gro g96 pdb cpt |
| **-s** | topol.tpr | Input | Structure+mass(db): tpr tpb tpa gro g96 pdb |
| **-n** | index.ndx | Input, Opt. | Index file |
| **-q** | eiwit.pdb | Input, Opt. | Protein data bank file |
| **-oq** | bfac.pdb | Output, Opt. | Protein data bank file |
| **-ox** | xaver.pdb | Output, Opt. | Protein data bank file |
| **-o** | rmsf.xvg | Output | xvgr/xmgr file |
| **-od** | rmsdev.xvg | Output, Opt. | xvgr/xmgr file |
| **-oc** | correl.xvg | Output, Opt. | xvgr/xmgr file |
| **-dir** | rmsf.log | Output, Opt. | Log file |

### Other options

| option | type | default | description |
| --- | --- | --- | --- |
| **-[no]h** | bool | no | Print help info and quit || **-[no]version** | bool | no | Print version info and quit || **-nice** | int | 19 | Set the nicelevel || **-b** | time | 0 | First frame (ps) to read from trajectory || **-e** | time | 0 | Last frame (ps) to read from trajectory || **-dt** | time | 0 | Only use frame when t MOD dt = first time (ps) || **-[no]w** | bool | no | View output .xvg, .xpm, .eps and .pdb files || **-xvg** | enum | xmgrace | xvg plot formatting: xmgrace, xmgr or none || **-[no]res** | bool | no | Calculate averages for each residue || **-[no]aniso** | bool | no | Compute anisotropic termperature factors || **-[no]fit** | bool | yes | Do a least squares superposition before computing RMSF. Without this you must make sure that the reference structure and the trajectory match. |

---

http://www.gromacs.org  
gromacs@gromacs.org
